# DUB1 suppresses Hippo signaling by modulating TAZ protein expression in gastric cancer

**DOI:** 10.1186/s13046-022-02410-5

**Published:** 2022-07-12

**Authors:** Dehai Wang, Zhongbo Li, Xin Li, Cheng Yan, Huijie Yang, Ting Zhuang, Xiao Wang, Yifeng Zang, Ziping Liu, Tianshi Wang, Rixia Jiang, Peng Su, Jian Zhu, Yinlu Ding

**Affiliations:** 1 Department of General Surgery, The Second Hospital, Cheeloo College of Medicine, Shandong University, Shandong Province, People’s Republic of China; 2 Xinxiang Key Laboratory of Tumor Migration and Invasion Precision Medicine, School of Laboratory Medicine, Xinxiang Medical University, Xinxiang, 453003 Henan Province People’s Republic of China; 3 School of Medicine, Xinxiang University, Xinxiang, Henan People’s Republic of China; 4 Department of Burns and Plastic Surgery, The Second Hospital, Cheeloo College of Medicine, Shandong University, Shandong Province, People’s Republic of China; 5 Department of Hepatobiliary Surgery, The Second Hospital, Cheeloo College of Medicine, Shandong University, Shandong Province, People’s Republic of China; 6 Department of Pathology, Shandong University Qilu Hospital, Cheeloo College of Medicine, Shandong University, Shandong Province, People’s Republic of China

**Keywords:** DUB1, TAZ, Gastric cancer, Deubiquitination, Stabilize

## Abstract

**Background:**

The Hippo pathway functions as a tumor suppressor pathway in human cancers, while dysfunction of the Hippo pathway is frequently observed in malignancies. Although YAP/TAZ activity is tightly controlled by the phosphorylation cascade of the MST-LATS-YAP/TAZ axis, it is still unclear why the YAP/TAZ proteins are activated in human cancers despite Hippo pathway activation. Recent studies have suggested that in addition to phosphorylation, several other posttranslational modifications, including ubiquitination, also play critical roles in modulating TAZ function.

**Methods:**

We used several gastric cancer cell lines and performed western blot analysis, real-time PCR, immunoprecipitation assays, and in vitro ubiquitination assays and established a xenograft mouse model.

**Results:**

Here, by screening a DUB (deubiquitinase) siRNA library, we discovered that DUB1 functions as a critical modulator that facilitates gastric cancer stemness and progression by deubiquitinating and activating the TAZ protein. We also found that DUB1 expression was elevated in gastric cancer and that elevated DUB1 expression correlated with TAZ activation and poor survival. DUB1 associates with the TAZ protein and deubiquitinates TAZ at several lysine residues, which subsequently stabilizes TAZ and facilitates its function.

**Conclusions:**

Our study revealed a novel deubiquitinase in the Hippo/TAZ axis and identified one possible therapeutic target for Hippo-driven gastric cancer.

**Supplementary Information:**

The online version contains supplementary material available at 10.1186/s13046-022-02410-5.

## Highlights

1. DUB1 is an important modulator of Hippo signaling in gastric cancer.

2. DUB1 expression is elevated in human gastric cancer samples and correlates with poor survival in gastric cancer.

3. DUB1 facilitates gastric cancer progression via the Hippo/TAZ axis.

4. DUB1 associates with TAZ and directly inhibits YAP polyubiquitination and degradation.

**Supplementary Information:**

The online version contains supplementary material available at 10.1186/s13046-022-02410-5.

## Background

Gastric cancer has the fifth highest incidence and is the fourth leading cause of cancer-related mortality in China [1]. According to epidemiological reports in 2020, more than one million new gastric cancer cases were diagnosed, more than half of which were in China [2]. The process of gastric cancer carcinogenesis is a multistep transformation of several types of gastric lesions, including gastritis, intestinal metaplasias and epithelial neoplasias [3]. Due to its insidious onset and mild symptoms, most gastric cancer patients are diagnosed at an advanced stage, when the 5-year survival rate is only 35% [4]. Several biomedical studies have shown the involvement of *Helicobacter pylori* infection and chronic inflammation in gastric cancer development [5]; however, the detailed mechanisms underlying gastric cancer progression are still unclear. Thus, it is urgent and necessary to develop novel biomarkers and therapeutic targets for gastric cancer.

Several studies have established a potential link between Hippo signaling and gastric cancer [6]. The Hippo signaling pathway plays essential roles in several biological processes, including organ size control, tissue homeostasis, carcinogenesis and immune responses [7]. Activation of Hippo signaling is controlled by a phosphorylation cascade, in which the mammalian Hippo kinases MST1/2 phosphorylate the LATS1/2 kinases, while phosphorylated LATS1/2 promote the phosphorylation of the YAP/TAZ proteins, leading to their cytosolic localization and degradation [8]. If Hippo signaling is inactivated, YAP/TAZ are unphosphorylated, translocate into the nucleus and interact with several transcription factors to promote the expression of their target genes [9]. Dysregulation of Hippo signaling has been observed in several human malignancies. While overexpression of YAP/TAZ has been found in gastric cancer, functional blockade of YAP/TAZ can inhibit gastric cancer progression in mouse models of *H. pylori*-induced gastric cancer [10]. However, although many efforts have been made to pharmaceutically target Hippo signaling, the clinical application of these strategies is still immature.

TAZ (Transcriptional Co-Activator With PDZ-Binding Motif) is a downstream effector of Hippo signaling and a transcriptional cofactor for several transcription factors, including TEADs [11]. Several studies have suggested that TAZ could be a critical factor for carcinogenesis in humans. For example, molecular studies revealed that TAZ activation can transactivate several signaling pathways, such as the Wnt and Hedgehog signaling pathways [12, 13]. In addition, the TAZ protein level was found to be elevated in samples of several human cancers, including breast cancer and gastric cancer, and a high TAZ expression level was correlated with poor prognosis [14–16]. Based on this finding, targeting TAZ, which subsequently blocks its transactivation effects, could be a promising strategy. Thus, as TAZ is an obvious candidate component of the ubiquitin–proteasome system, targeting its protein stability could be utilized to restore the tumor-suppressive function of Hippo signaling and inhibit gastric cancer progression.

The regulation of protein stability is controlled by the balance of E3 ubiquitin ligases and deubiquitinases, which is crucial for maintaining normal physiological homeostasis [17, 18]. Disrupted regulation of protein ubiquitination could contribute to several human diseases, including cancers [19]. We and other groups discovered several E3 ubiquitin ligases that exert their effects on Hippo pathway effectors and modulate Hippo signaling activity and cancer progression [20–25]. Although there are studies reporting the involvement of several deubiquitinases in modulating Hippo signaling, it is still largely unclear which of these deubiquitinases exerts important impacts on Hippo signaling. By screening a DUB (deubiquitinase) siRNA library, we identified DUB1 as a critical modulator of Hippo pathway activity in gastric cancer. DUB1 expression was increased in gastric cancer samples, correlated with poor prognosis in gastric cancer patients and correlated with TAZ expression. DUB1 was found to facilitate gastric cancer progression through the Hippo/TAZ axis. DUB1 was found to associate with TAZ in the nucleus and inhibit K48-linked ubiquitination and degradation. Hence, DUB1 functions to enhance Hippo signaling activity in gastric cancer, which could be a promising diagnostic and therapeutic marker in gastric cancer.

## Materials and Methods

### Cell culture

Gastric cancer cell lines (AGS and MGC803) were cultured in DMEM (Thermo Fisher Scientific, Cat: 11,965,092) containing 10% fetal bovine serum (Biological Industries, BISH0744) and 1% penicillin/streptomycin (Invitrogen). HEK-293 cells were cultured in DMEM supplemented with 10% FBS (Biological Industries, BISH0744) and 1% penicillin/streptomycin. AGS and BCG803 gastric cancer cells were authenticated by short tandem repeat (STR) profiling. The STR profile of our AGS cells was found to be 100% consistent with the STR data of AGS cells from the China Infrastructure of Cell Line Resources. MGC803 cell STR profiling data are not accessible in public databases, including ATCC. Cells were regularly tested for mycoplasma contamination using a LookOut Mycoplasma PCR Detection Kit (MP0035, Sigma) and were used only when negative.

### RNA isolation and quantitative real-time PCR (qRT–PCR)

Total RNA was extracted with an RNeasy Plus Mini Kit (Tiangen, DP451) following the manufacturer’s specifications. Reverse transcription was performed using HiScript II Q RT SuperMix (Vazyme, R223-01). qRT–PCR was carried out using SYBR qPCR Master Mix (Vazyme, Q511-02) and a 7500 Fast Real-Time PCR System (Applied Biosystems, Singapore). 36B4 was used as an internal control. The sequences of the primers used for qPCR were as follows: 36B4 F: GGC GAC CTG GAA GTC CAA CT; R: CCA TCA GCA CCA CAG CCT TC. CTGF F: CTC GCG GCT TAC CGA CTG; R: GGC TCT GCT TCT CTA GCC TG. CYR61 F: AGC AGC CTG AAA AAG GGC AA; R: AGC CTG TAG AAG GGA AAC GC. DUB1 F: GAG GCC GGG GCT CTG A; R: ACT GGG ATG TGC AGA CTT GG. TAZ F: AGA GTC GGG TCG GGA TTT GT; R: AGG CCG GAT TCA TCT TCT GGG.

### Plasmids and siRNA

The DUB1 and TAZ plasmids were acquired from HANBIO Company (https://www.hanbio.net). The DUB1 and TAZ deletion constructs were subcloned from the full-length plasmid DNA. The HA-K48 and HA-Ub plasmids were used in a previous study. Lipofectamine 2000 (1,662,298, Invitrogen) was used for plasmid transfection. Small interfering RNAs were used for knockdown of specific genes. The DUB1 siRNA sequences were as follows: (1) CCG GCA AGC UGC GAA UAU UTT and AAU AUU CGC AGC UUG CCG GTT; and (2) GCA CAC CAC UGA AGA GAU UTT and AAU CUC UUC AGU GGU GUG CTT. The negative control siRNA sequences were as follows: UUC UCC GAA CGU GUC ACG UTT and ACG UGA CAC GUU CGG AGA ATT. RNAiMAX reagent (13,778,150, Invitrogen) was used for siRNA transfection. For lentiviral DUB1 silencing, shDUB1 was inserted into the vector pLKO.1, which was cotransfected with the pMD2.G envelope plasmid and psPAX2 packaging plasmid into HEK293 cells. The DUB1 shRNA-expressing lentivirus was harvested after 48 h. Gastric cancer cells were incubated with 2 ml of antibiotic-free medium containing 200 µl of lentiviral suspension.

### Western blot analysis

Standard western blotting techniques were utilized to analyze relative protein expression in cells. The following antibodies were used for western blot analysis: anti-Flag-M2 (A8592, Sigma, 1:1000), anti-HA (2,013,819,001, Roche, 1:1000), anti-Myc (9E10, Santa Cruz, 1:1000), anti-GAPDH (0411, Santa Cruz, 1:1000), anti-TAZ (Cell Signaling Technology, CST83699, 1:1000), and anti-DUB1 (Sigma, HPA12082, 1:1000). Protein signals were detected with an ECL kit (Millipore Co., Billerica, Massachusetts, USA).

### Luciferase reporter assays

For TEAD luciferase activity assays, MGC803 and AGS cells expressing siDUB1 or siControl were transfected with the TEAD luciferase reporter vector for 24 h. Cells were then harvested for assays. Luciferase reporter assays were performed using a dual luciferase assay kit (Promega). The pRL-null vector expressing Renilla luciferase (Promega) was used as the internal control for normalization of the transfection efficiency.

### Wound healing and Transwell assays

For the wound healing assay, MGC803 and AGS cells expressing siDUB1 or siControl were seeded in a 6-well plate until confluent, and a wound was then made by scratching with a sterile tip. Images of the cells were acquired at the indicated time points after wounding. The distance between the two edges of the scratch wound was measured using ImageJ software. The Transwell system (8 μm pore size, Corning) was employed for cell migration and invasion assays. For invasion assays, the membranes in the upper chambers were coated with Matrigel (BD Biocoat, USA). After 24 h, the gastric cancer cells that had migrated to the bottom surface of the insert membrane were fixed, stained with crystal violet and counted under a 20 × objective. The experiments were performed in triplicate.

### Cycloheximide assay

MGC803 cells were transfected with siDUB1 or siControl for 24 h. After that, cycloheximide was added to the culture medium at a final concentration of 100 μmol/L. Cell lysates were collected at 0, 3, 6 and 9 h after cycloheximide treatment. HEK293 cells were transfected with 2 µg of the Flag-DUB1 or Flag vector. After 24 h, the cells were treated with cycloheximide at a final concentration of 100 μmol/L. Cell lysates were collected at 0, 3, 6 and 9 h after cycloheximide treatment.

### Immunofluorescence (IF) staining

MGC803 cells on coverslips were fixed with 4% paraformaldehyde and incubated with primary antibodies against DUB1 (Sigma, A300-940A) and TAZ (CST, 71,192) at 4 °C overnight. After that, the cells were washed with PBS. Then, the cells were incubated with a fluorophore-conjugated secondary antibody (Invitrogen, Carlsbad, CA). Finally, the cells were counterstained with DAPI (Life Technology). Images of stained cells were acquired with a confocal laser scanning microscope (Leica TCS SP8 STED). The -integrated fluorescence density was measured by ImageJ software.

### Clone formation assays

MGC803 and AGS cells were seeded in six-well plates overnight and treated with 50 nM DUB1 siRNA or 50 nM siControl. After twenty-four hours, the gastric cancer cells were washed with PBS, trypsinized and plated at a low density (5000 cells/well in a six-well plate). The cells were cultured for 10 days, and the medium was refreshed every two or three days. Colonies were stained with crystal violet. The colonies in a given area were counted for each condition.

### Co-IP assay

The coimmunoprecipitation assay was performed as previously described. Total lysates of MGC803 cells were collected and precleared with rabbit IgG for 2 h and were subsequently immunoprecipitated with an anti-DUB1 antibody (Sigma, A300-940A) overnight, with rabbit IgG (Santa Cruz) as the negative control. Bound proteins were analyzed by western blotting with an anti-TAZ antibody (CST, 71,192). For the overexpression experiment, HEK293 cells were transfected with 5 µg of Flag-DUB1 (full-length or domain deletion mutants) and Myc-TAZ (full-length or domain deletion mutants) in 10 cm dishes. Cell lysates were precleared with IgG and subsequently incubated with an anti-Flag-M2 antibody (A8592, Sigma, 1:1000), with mouse IgG as the negative control. Bound proteins were analyzed by western blotting.

### Pull-down assay

TAZ protein was purchased from NOVUS Biological Company (Cat: NBP2-22,949). GST protein and GST-fusion DUB1 truncate domains expression plasmids were expressed in E coli and purified by GST agarose beads. The mixture of TAZ protein and GST-fusion DUB1 domains were incubated at 4 degree for 30 min. The resin was washes with PBS containing 30 mM imidazole. Then the mixture was washed with PBS containing 0.01% Triton 100. The bound proteins were eluted and subject to western blot analysis.

### In vitro ubiquitination assays

For in vitro ubiquitination assays, cells were separately transfected with vectors, including the Myc-TAZ, Flag-DUB1 and HA-Ub expression vectors, for 24 h. Cells were then treated with MG132 (10 μM) for 6 h, and the level of Myc-TAZ ubiquitination was determined by IP with an anti-HA antibody (2,013,819,001, Roche, 1:1000) followed by western blot analysis with an anti-Myc antibody (9E10, Santa Cruz, 1:1000).

### In vivo tumorigenesis assay

For the in vivo tumorigenesis assay, MGC803 cells were infected with shControl or shDUB1 lentivirus. After 48 h of infection, the cells were treated with 1 µg/ml puromycin for 3 days. MGC803 cells (2 × 10^6^) were injected into the right dorsal flanks of 4-week-old female BALB/c nude mice. Tumor formation in the nude mice was monitored over a 4-week period. The tumor volume was calculated with the following equation: tumor volume = 0.5 × length × width^2^. The mice were sacrificed five weeks after injection. After the mice were sacrificed, the tumors were weighed and photographed. The experiments were performed under the protocols approved by the ethics committee of Xinxiang Medical University.

### Cell proliferation assay

MGC803 and AGS cells were transfected with siDUB1 or siControl in 24-well plates. Twenty-four hours after transfection, the cells were counted, and 4000 cells were seeded into 96-well plates. Relative cell viability was measured at the indicated time points. Cell numbers were determined using CCK8 cell proliferation reagent and measuring the absorbance at 450 nm. Cell proliferation was further analyzed by EdU incorporation and flow cytometry assays. The numbers of gastric cancer cells were determined by using a 5-ethynyl-20-deoxyuridine (EdU) assay kit (RiboBio, Guangzhou, China). For quantitative analysis of images, each data point was considered to represent the fluorescence-positive area calculated from a minimum of five randomly selected fields from three individual experiments. EdU incorporation and FACS assays were performed according to the manufacturer’s instructions. The experiments were performed in triplicate. For cell cycle analysis, MGC823 cells were transfected with 50 nM siDUB1 or siControl. After 24 h, the cells were fixed with 70% ethanol and stained with propidium iodide. Twenty-four hours post transfection, the cells were stained with propidium iodide and annexin V. A BD LSR FACS instrument was used to measure the fluorescence intensity.

### Tissue microarray (TMA) and immunohistochemical (IHC) analyses

One hundred paraffin-embedded gastric cancer samples were acquired from Shanghai Outdo Biotech Company (http://www.superchip.com.cn). All gastric tumor samples were examined by pathology specialists. The pathological grade and lymph node metastasis status of each sample were obtained from Shanghai Outdo Biotech Company. The usage of the samples was approved by Shanghai Outdo Biotech Company with written informed consent from all patients. Specific antibodies against TAZ (CST, 71,192) and DUB1 (Sigma, HPA12082) were used to detect the staining density in human samples. Scores were calculated based on the staining intensity and percentage of positive tumor cells in the whole tissues, which were evaluated according to the Fromowitz standard. The staining intensity was graded as follows: no staining, 0; weak positive staining, 1; moderate positive staining, 2 and strong positive staining, 3. The percentage of positive cells was divided into four categories: 0–25% staining, 1; 26–50% staining, 2; 51–75% staining, 3; and 76–100% staining, 4. Staining scores of 1–2 were regarded as indicating low expression, while staining scores of 3–4 were regarded as indicating high expression. All staining was assessed at 200X magnification, and at least three fields from each core were counted.

### RNA sequencing and data analysis

Global gene expression analysis (siControl and siDUB1) was performed with the RNA sequencing platform from BGI (Beijing Genomics Institute). The RNA sequencing data have been deposited in the Gene Expression Omnibus (GEO) database (accession number: GSE143947). Differentially expressed genes (*P* < 0.01 and fold change > 2) were subjected to Ingenuity Pathway Analysis (IPA). For gene set enrichment analysis of RNA-seq data, the conserved Hippo signature gene sets were used and downloaded from Molecular Signatures Database v7.4. GSEA was implemented using GSEA 4.1.0 software with default parameters. A volcano plot was generated using the ‘ggplot2’ package in R (threshold *P* < 0.05 and fold change > 1.5).

### In vitro deubiquitination assay

The proteins were over-expressed in HEK293 cells and immunoprecipitated with antibodies. Ubiquitination was analyzed with an ubiquitination kit (Boston Biochem) following protocols recommended by the manufacturer. Recombinant proteins were mixing with 20X E1 Enzyme, 10X Mg2 + -ATP Solution, 10X Ubiquitin Solution, 1ug E2 Enzyme (UbcH7, Boston Biochem; UBE2D1, Sino Biological Inc.) in a final volume of 20 ul reaction buffer. The reaction was carried out at 37 °C for 1 h and products were analyzed by western-blot assays with anti-TAZ antibody.

### The precise sites of protein for deubiquitination

HEK293 cells were transfected with indicated WT vectors or mutation vectors for ubiquitination assays. The poly-ubiquitinated TAZ was detected through the analysis of western blotting. After 24 h, cells were then treated with MG132 (10 μM) for 6 h, and the level of Myc-TAZ ubiquitination was determined by IP with an anti-HA antibody (2,013,819,001, Roche, 1:1000) followed by western blot analysis with an anti-Myc antibody (9E10, Santa Cruz, 1:1000).

### Analysis of TCGA data and progression-free survival data

Gene expression data for 385 gastric cancer patients in TCGA were downloaded from the website (http://gepia.cancer-pku.cn/index.html). The DUB1 mRNA levels in normal gastric tissue and gastric cancer tissues of different stages were generated with GEPIA online software. The progression-free survival (PFS) data of patients stratified by TAZ and DUB1 expression were generated from the KMPLOT online analysis database (https://kmplot.com). The Affy IDs of TAZ and DUB1 were 202133_at and 227093_at, respectively. The PFS survival data of gastric cancer patients stratified by DUB1 and TAZ expression were generated from the KMPLOT database.

### Statistical analysis

No specific statistical tests were used to predetermine the sample size. Statistical analysis was performed using GraphPad Prism 7 software or SPSS version 23.0. Data are expressed as the mean ± s.e.m. values. Differences between two independent groups were evaluated with Student’s t test. The Kaplan − Meier method with the log-rank test was applied for survival analysis. Differences were considered to be statistically significant when *P* < 0.05 (*P < 0.01; **P < 0.001).

## Results

### DUB1 is elevated in human gastric cancer and correlates with Hippo signaling activity on a -genome-wide scale

**Fig. 1:**
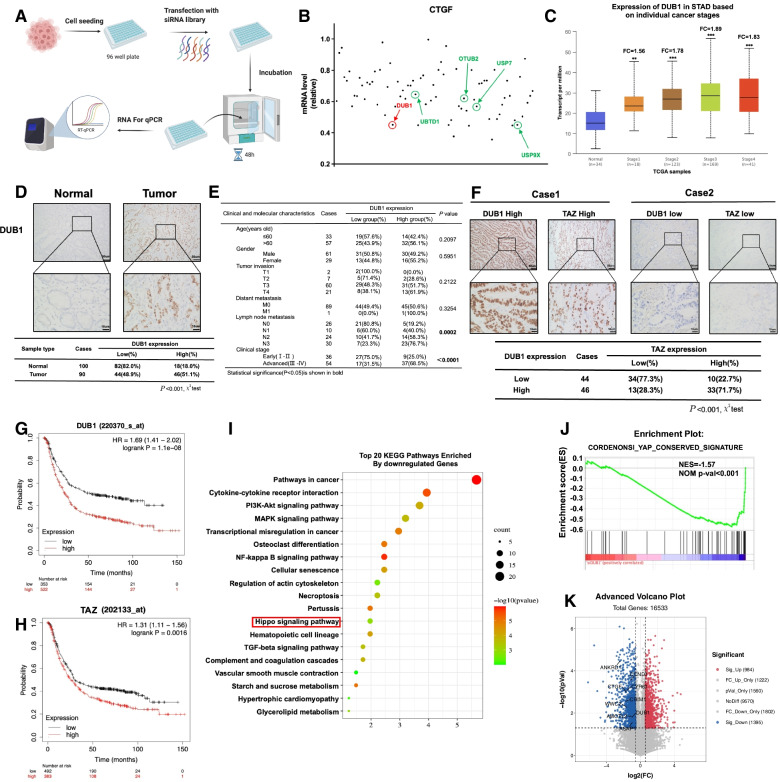
DUB1 expression is elevated in human gastric cancer and correlates with Hippo signaling activity on a whole-genome scale **A:** Flowchart of the siRNA screening process to identify novel deubiquitinases involved in modulating Hippo signaling. HEK293 cells were seeded in 96-well plates. siRNA for one deubiquitinase was transfected into each well and incubated for 48 h. The cells were used for RNA extraction and qPCR analysis of CTGF expression. **B:** The relative mRNA level of CTGF determined by deubiquitinase siRNA screening. The green dots represent the DUBs already reported to be linked to Hippo signaling in a previous study. The red dots represent the CTGF mRNA levels after DUB1 depletion.**C:** The DUB1 expression level was significantly elevated in gastric cancer tissues of different stages compared with normal gastric tissue. Data were generated from the TCGA database (https://www.genome.gov/). **D:** DUB1 protein expression was significantly increased in gastric tumor tissues compared with the corresponding adjacent nontumor tissues, as determined by immunohistochemical staining. **E:** Correlations between the DUB1 expression level in gastric tumor samples and clinicopathological characteristics of the corresponding patients. Data analysis revealed that DUB1 expression was correlated with lymph node metastasis and advanced tumor stage (*P* = 0.0002 and *P* < 0.0001, respectively). **F:** DUB1 expression was significantly correlated with increased TAZ expression in gastric cancer specimens, as determined by IHC staining. **G:** Kaplan − Meier analysis revealed that DUB1 expression was related to poorer progression-free survival in gastric cancer patients. *P* < 0.001, log-rank test. **H:** Kaplan − Meier analysis revealed that TAZ expression was related to poorer progression-free survival in gastric cancer patients. *P* = 0.0016, log-rank test. **I:** Top 20 KEGG pathways significantly depleted (top) or enriched (bottom) in MCG803 cells treated with siDUB1. Pathway enrichment analysis was performed with differentially regulated genes identified with the threshold criteria *P* < 0.001 and fold change > 2. Cells were treated with vehicle or 50 nM siDUB1 for 48 h. Total mRNA was extracted for RNA sequencing analysis. *n* = 3. **J:** Gene set enrichment analysis (GSEA) showed depletion of Hippo pathway signature genes in MGC803 cells treated with DUB1 siRNA. **K:** Volcano plot showing that DUB1 depletion inhibited the expression of the Hippo pathway signature genes (red) in MCG823 cells. Threshold criteria: *P* < 0.05 and fold change > 1.5

Since these studies aimed to identify novel deubiquitinating enzymes involved in the regulation of Hippo signaling, we carried out a DUB (deubiquitinase) siRNA screen with a DUB siRNA library (Dharmacon Company, Cat: G104705). Since HEK293 cells are widely used for Hippo signaling studies and can be transfected with high efficiency, we utilized HEK293 cells for initial screening. Since CTGF is regarded as one of the most classical target genes, we used CTGF as the endpoint indicator of Hippo signaling activity (Fig. 1A). The siRNA screen coupled with qPCR revealed several confirmed DUBs, such as USP9X and USP7, which were proven to regulate the Hippo pathway in previous publications (Supplementary Table 1). However, we discovered that the unreported deubiquitinase DUB1 could also be an important regulator in the Hippo pathway (Fig. 1B). We further analyzed the DUB1 mRNA level in gastric cancer patients. TCGA data showed that DUB1 expression was significantly elevated in gastric cancer samples compared with normal gastric tissue samples (Fig. 1C). The immunohistochemical data showed that DUB1 expression was significantly higher in gastric cancer samples, and DUB1 expression was correlated with lymph node metastasis and higher clinical stage (*P* < 0.001, *P* < 0.001, and *P* < 0.001, respectively; Fig. 1D-1E). Interestingly, the protein level of DUB1 was positively correlated with the TAZ expression level in gastric cancer samples (*P* < 0.001, Fig. 1F). The TCGA data analysis showed that DUB1 expression positively correlated with several classical Hippo target gene expression, including CTGF, MYC, AJUBA, AMOTL2, TOP2A and ETV5 (Supplementary Fig. 1). Analysis of prognostic data showed that both DUB1 and TAZ were correlated with poor survival in gastric cancer patients (Fig. 1G-1H). While considering both DUB1 and CTGF expression, the prognosis was poor in High^CTGF+DUB1^ compared with Low^CTGF+DUB1^ group in gastric cancer patients (Median survival: 28.8 month vs 89.4 month; *P* < 0.0001) (Supplementary Fig. 2F-2G). To investigate the function of DUB1 in gastric cancer via an unbiased approach, we depleted DUB1 in MGC803 cells for whole-genome expression analysis. KEGG pathway enrichment analysis indicated that DUB1 depletion could affect several cancer-related pathways, including the Hippo signaling and PI3K/AKT pathways (Fig. 1I). GSEA indicated that DUB1 depletion significantly inhibited Hippo signaling activity (Fig. 1J). The volcano plot showed that DUB1 silencing dramatically inhibited the expression of classical Hippo target genes, including CTGF, CYR61 and ANKRD1 (Fig. 1K).

### DUB1 facilitates Hippo/TAZ axis activity in gastric cancer cells

**Fig. 2:**
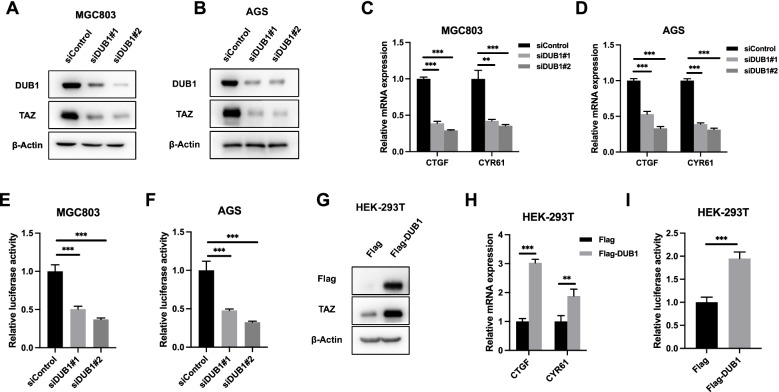
DUB1 facilitates Hippo/TAZ axis activity in gastric cancer cells **A:** DUB1 depletion reduced the TAZ protein level in MGC803 cells. MGC803 cells were transfected with siControl or siDUB1. After 48 h, the cells were harvested for western blot analysis. DUB1 and TAZ protein levels were determined by Western blotting. Actin was used as the internal control. **B:** DUB1 depletion reduced the TAZ protein level in AGS cells. AGS cells were transfected with siControl or siDUB1. After 48 h, the cells were harvested for western blot analysis. DUB1 and TAZ protein levels were determined by Western blotting. Actin was used as the internal control. **C:** DUB1 depletion decreased Hippo target gene expression in MGC803 cells. MGC803 cells were transfected with siControl or siDUB1. After 48 h, total RNA was extracted for gene expression analysis. Each group was tested in triplicate. *P < 0.05, **P < 0.01, ***P < 0.001 for comparisons of target gene expression. **D:** DUB1 depletion decreased Hippo target gene expression in AGS cells. AGS cells were transfected with siControl or siDUB1. After 48 h, total RNA was extracted for gene expression analysis. Each group was tested in triplicate. *P < 0.05, **P < 0.01, ***P < 0.001 for comparisons of target gene expression. **E:** DUB1 depletion decreased TEAD luciferase activity in MGC803 cells. MGC803 cells were transfected with siControl or siDUB1. After 24 h, the cells were transfected with TEAD luciferase reporter plasmids. After another 24 h, the cells were harvested for luciferase activity analysis. **F:** DUB1 depletion decreased TEAD luciferase activity in AGS cells. AGS cells were transfected with siControl or siDUB1. After 24 h, the cells were transfected with TEAD luciferase reporter plasmids. After another 24 h, the cells were harvested for luciferase activity analysis. **G:** DUB1 overexpression increased the TAZ protein level in HEK293 cells. HEK293 cells were transfected with 2 µg of the Flag-DUB1 or Flag vector. After 48 h, the cells were harvested for western blot analysis. DUB1 and TAZ protein levels were determined by Western blotting. Actin was used as the internal control. **H:** DUB1 overexpression increased Hippo target gene expression in HEK293 cells. HEK293 cells were transfected with 2 µg of the Flag-DUB1 or Flag vector. After 48 h, total RNA was extracted for gene expression analysis. Each group was tested in triplicate. *P < 0.05, **P < 0.01, ***P < 0.001 for comparisons of target gene expression. **I:** DUB1 overexpression increased TEAD luciferase activity in HEK293 cells. HEK293 cells were transfected with 2 µg of the Flag-DUB1 or Flag vector. After 24 h, the cells were transfected with TEAD luciferase reporter plasmids. After 24 h, the cells were harvested for luciferase activity analysis

We further utilized two independent siRNAs for DUB1 to avoid off-target effects. Western blot analysis showed that DUB1 depletion did not affect the YAP protein level in AGS and MGC803 cells (data not shown). However, DUB1 depletion dramatically decreased the TAZ protein level in AGS and MGC803 cells (Fig. 2A-2B). We examined Hippo target gene expression in gastric cancer cells. We found that DUB1 depletion inhibited the expression of Hippo target genes, including CTGF and CYR61, in AGS and MGC803 cells (Fig. 2C-2D). We further evaluated the effect of DUB1 on Hippo signaling activity. The luciferase reporter assay showed that DUB1 silencing inhibited TEAD response element activity in AGS and MGC803 cells (Fig. 2E-2F). Consistent with these results, HEK293 cells with transient DUB1 overexpression showed an increased TAZ protein level, increased Hippo target gene expression (CTGF and CYR61) and increased luciferase activity of TEAD response elements (Fig. 2G-2I).

### DUB1 depletion inhibits gastric cancer progression in vivo and in vitro.

**Fig. 3:**
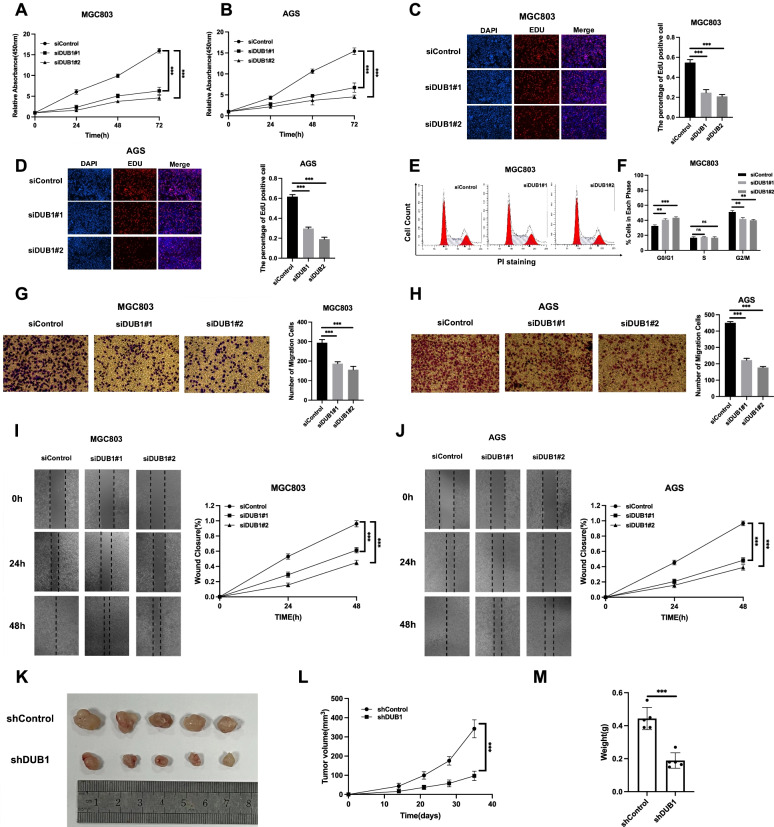
DUB1 depletion inhibits gastric cancer progression in vivo and in vitro. **A-B:** Depletion of DUB1 inhibited the proliferation of gastric cancer cells. MGC803 and AGS cells were transfected with siControl or siDUB1. Two different siRNAs were used. After 24 h, a WST-1 assay was used to determine the cellular metabolic activity at the indicated time points after transfection. Experiments were performed in triplicate. *P < 0.05, **P < 0.01, ***P < 0.001 for comparisons of cell growth. **C-D:** DUB1 depletion reduced the number of EdU-positive gastric cancer cells. MGC803 and AGS cells were transfected with siControl or siDUB1. After 24 h, EdU was added to the medium for 2 h of incubation. The absolute cell number was determined to indicate cell proliferation activity. **E–F:** Cell cycle analysis was performed to assess the effect of DUB1 silencing on MGC803 cells. MGC803 cells were transfected with 50 nM DUB1 siRNA or 50 nM control siRNA. After 24 h, the cells were harvested, fixed with 70% ethanol and stained with propidium iodide. The cells were subjected to FACS analysis. Experiments were performed in triplicate. *P < 0.05, **P < 0.01, ***P < 0.001 for comparisons of cell proportions. Representative histograms and cell cycle phase distribution plots are shown in Fig. 3E and 3F, respectively. **G-H:** DUB1 promoted the migration of MGC803 and AGS gastric cancer cells. MGC803 and AGS cells were transfected with siControl or siDUB1. After 24 h, Transwell assays were used to evaluate the migratory capacity. The cell number was determined, and the data are presented as the means ± SDs. **P < 0.01, ***P < 0.001 (Student’s t test). **I-J:** Wound healing assay of MGC803 and AGS cells with DUB1 depletion or siControl transfection. Quantification of wound closure at the indicated time points. The data are presented as the means ± SDs. **P < 0.01, ***P < 0.001 (Student’s t test). **K-M:** DUB1 depletion inhibited gastric tumor growth in vivo. MGC803 cells were stably transduced with lentiviral vectors expressing scrambled shRNA or DUB1 shRNA. These MGC803 cells (2 × 10^6^) were injected into the right dorsal flanks of 4-week-old female BALB/c nude mice. Tumor formation in the nude mice was monitored over a 4-week period. The tumor volume was calculated with the following equation: tumor volume = 0.5 × length × width^2^. Mice were sacrificed five weeks after tumor cell injection. Tumor growth curves, weights and photographs are shown in Panels K, L and M, respectively

To investigate the impact of DUB1 on the gastric cancer phenotype, we depleted DUB1 in MGC803 and AGS cells. The CCK8 assay indicated that DUB1 depletion significantly inhibited gastric cancer cell growth (Fig. 3A-3B). In addition, the EdU incorporation assay showed that DUB1 silencing dramatically decreased the number of EdU-positive MGC803 and AGS cells (Fig. 3C-3D). Cell cycle analysis by PI staining and FACS showed that DUB1 depletion significantly increased the proportion of G1-phase cells (Fig. 3E-3F). In addition, the Transwell assay indicated that DUB1 was necessary for the invasive capacity of AGS and MGC803 cells (Fig. 3G-3H). The wound healing assay showed that DUB1 depletion decreased the migration speed of AGS and MGC803 cells (Fig.  3I-3J). In the in vivo tumor growth assay, stable silencing of DUB1 in MG803 cells inhibited tumor growth in the xenograft mouse model (Fig. 3K-3M).

### DUB1 controls gastric cancer progression via the Hippo/TAZ axis

**Fig. 4:**
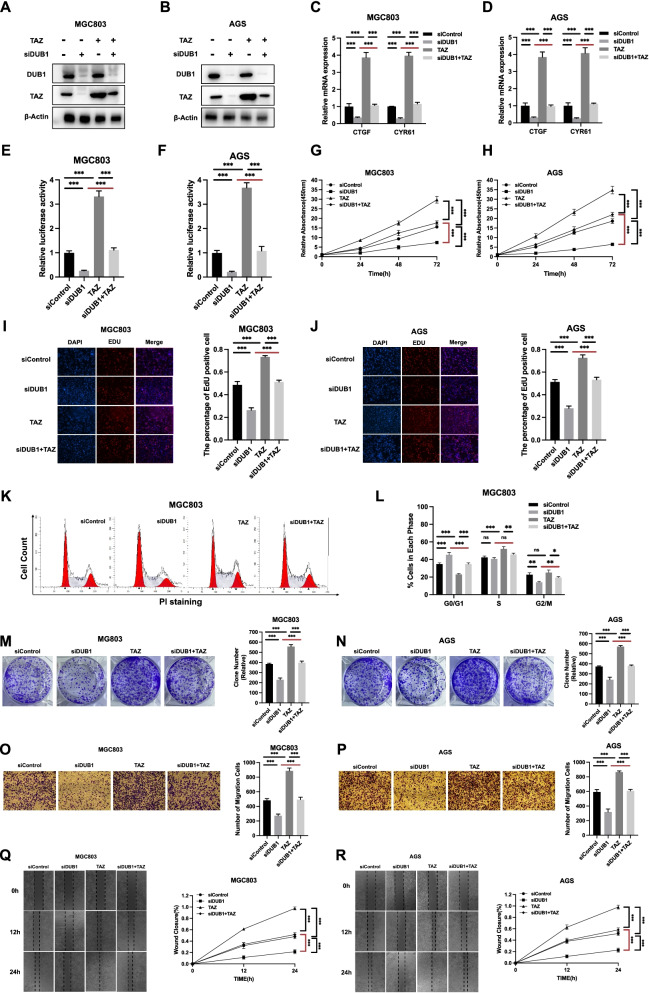
DUB1 controls gastric cancer progression via the Hippo/TAZ axis **A:** DUB1 depletion decreased the TAZ protein level, and this effect was reversed by TAZ overexpression. MGC803 cells were transfected with siControl or siDUB1. After 24 h, the cells were transfected with Flag-TAZ or siControl. After 48 h, the cells were harvested for western blot analysis. DUB1 and TAZ protein levels were determined by Western blotting. Actin was used as the internal control. **B:** DUB1 depletion decreased the TAZ protein level, and this effect was reversed by TAZ overexpression. AGS cells were transfected with siControl or siDUB1. After 24 h, the cells were transfected with Flag-TAZ or siControl. After 48 h, the cells were harvested for western blot analysis. DUB1 and TAZ protein levels were determined by Western blotting. Actin was used as the internal control. **C:** DUB1 depletion suppressed Hippo target gene expression, and this effect was reversed by TAZ overexpression. MGC803 cells were transfected with siControl or siDUB1. After 24 h, the cells were transfected with the Flag-TAZ or Flag vector. After 48 h, total RNA was extracted for gene expression analysis. Each group was tested in triplicate. *P < 0.05, **P < 0.01, ***P < 0.001 for comparisons of target gene expression. **D:** DUB1 depletion suppressed Hippo target gene expression, and this effect was reversed by TAZ overexpression. AGS cells were transfected with siControl or siDUB1. After 24 h, the cells were transfected with the Flag-TAZ or Flag vector. After 48 h, total RNA was extracted for gene expression analysis. Each group was tested in triplicate. *P < 0.05, **P < 0.01, ***P < 0.001 for comparisons of target gene expression. **E:** DUB1 depletion decreased TEAD luciferase activity in MGC803 cells, and this effect was reversed by TAZ overexpression. MGC803 cells were transfected with siControl or siDUB1. After 24 h, the cells were transfected with the Flag-TAZ or Flag vector. After 24 h, the cells were transfected with TEAD luciferase reporter plasmids. The cells were harvested for luciferase activity analysis. **F:** DUB1 depletion decreased TEAD luciferase activity in AGS cells, and this effect was reversed by TAZ overexpression. AGS cells were transfected with siControl or siDUB1. After 24 h, the cells were transfected with the Flag-TAZ or Flag vector. After 24 h, the cells were transfected with TEAD luciferase reporter plasmids. The cells were harvested for luciferase activity analysis. **G:** Cell growth inhibition induced by DUB1 silencing was rescued by TAZ overexpression in MGC803 cells. MGC803 cells were transfected with 50 nM DUB1 siRNA or 50 nM control siRNA. After 24 h, the cells were transfected with the Flag-TAZ or Flag vector. After 24 h, a CCK-8 assay was used to determine the cellular metabolic activity at the indicated time points after transfection. Experiments were performed in triplicate. *P < 0.05, **P < 0.01, ***P < 0.001 for comparisons of cell growth. **H:** Cell growth inhibition induced by DUB1 silencing was rescued by TAZ overexpression in AGS cells. AGS cells were transfected with 50 nM DUB1 siRNA or 50 nM control siRNA. After 24 h, the cells were transfected with the Flag-TAZ or Flag vector. After 24 h, a CCK-8 assay was used to determine the cellular metabolic activity at the indicated time points after transfection. Experiments were performed in triplicate. *P < 0.05, **P < 0.01, ***P < 0.001 for comparisons of cell growth. **I-J:** DUB1 depletion reduced the number of EdU-positive gastric cancer cells. This effect was further rescued by TAZ overexpression. MGC803 and AGS cells were transfected with siControl or siDUB1. After 24 h, the cells were transfected with the Flag-TAZ or Flag vector. EdU was added to the medium for 2 h of incubation. The absolute cell number was determined to indicate cell proliferation activity. **K-L:** Cell cycle arrest caused by DUB1 silencing was partially rescued by TAZ overexpression in MGC803 cells. MGC803 cells were transfected with 50 nM DUB1 siRNA or 50 nM control siRNA. After 24 h, the cells were transfected with the Flag-TAZ or Flag vector. The cells were harvested, fixed with 70% ethanol and stained with propidium iodide. The cells were subjected to FACS analysis. Experiments were performed in triplicate. *P < 0.05, **P < 0.01, ***P < 0.001 for comparisons of cell proportions. Representative histograms and cell cycle phase distribution plots are shown in Fig. 4 K and 4L, respectively. **M–N:** The reduction in the colony formation capacity induced by DUB1 silencing was rescued by TAZ overexpression in MGC803 and AGS cells. Gastric cancer cells were transfected with 50 nM DUB1 siRNA or 50 nM control siRNA. After 24 h, the cells were transfected with the Flag-TAZ or Flag vector. Quantification of colony formation is shown at the indicated time points. The data are presented as the means ± SDs. **P < 0.01, ***P < 0.001 (Student’s t test). **O-P:** DUB1 depletion decreased the invasive capacity of gastric cancer cells, and this effect was reversed by TAZ overexpression. MGC803 and AGS cells were transfected with 50 nM siControl or siDUB1. After 24 h, the cells were transfected with the Flag-TAZ or Flag vector. After another 24 h, the cancer cells were seeded into the chambers for the Transwell assay. The cell number was determined, and the data are presented as the means ± SDs. **P < 0.01, ***P < 0.001 (Student’s t test). **Q-R:** DUB1 depletion decreased the migratory capacity of gastric cancer cells, and this effect was reversed by TAZ overexpression. MGC803 and AGS cells were transfected with 50 nM siControl or siDUB1. After 24 h, the cells were transfected with the Flag-TAZ or Flag vector. After another 24 h, the cells were seeded in a 6-well plate until confluent, and a wound was then made by scratching with a sterile tip. Images of the cells were acquired at the indicated time points after scratching

To investigate the logical link between the gastric cancer phenotype and Hippo/TAZ signaling involving the function of DUB1, we carried out several rescue experiments. DUB1 depletion decreased the TAZ protein level, which was rescued by TAZ overexpression, in MGC803 and AGS cells (Fig. 4A-4B). The qPCR assay showed that DUB1 silencing decreased Hippo target gene expression, which was rescued by TAZ overexpression, in MGC803 and AGS cells (Fig. 4C-4D). The luciferase reporter assay showed that DUB1 silencing inhibited TEAD response element activity, which could be rescued by TAZ overexpression, in AGS and MGC803 cells (Fig. 4E-4F). The CCK8 assay showed that the inhibition of cell growth induced by DUB1 depletion was rescued by further TAZ overexpression in MGC803 and AGS cells (Fig. 4G-4H). The EdU staining assay showed that TAZ overexpression rescued EdU incorporation, which was decreased by DUB1 depletion, in cells Fig. 4I-4J. Cell cycle analysis showed that G1 arrest caused by DUB1 depletion was at least partially rescued by further TAZ overexpression (Fig. 4K-4L). The colony formation assay showed that the decrease in the colony number caused by DUB1 depletion was rescued by further TAZ overexpression in MGC803 and AGS cells (Fig. 4M). As shown in Fig. 4N-4O, DUB1 knockdown in gastric cancer cells inhibited cell invasion, which was at least partially rescued by further TAZ overexpression. The wound healing assay showed that DUB1 depletion reduced the migration speed, which was further rescued by TAZ overexpression, in MGC803 and AGS cells (Fig. 4P-4Q). These data indicate that DUB1 modulates Hippo signaling through the TAZ protein in gastric cancer.

### DUB1 associates with TAZ and modulates TAZ stability in gastric cancer cells

**Fig. 5:**
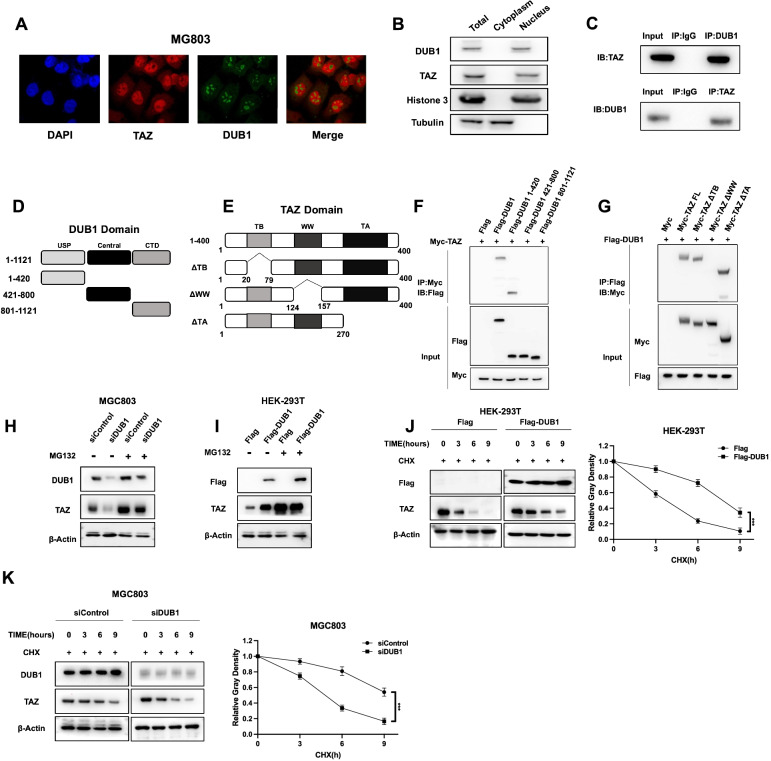
DUB1 associates with TAZ and modulates TAZ stability in gastric cancer cells **A:** Analysis of the intracellular localization of DUB1 and TAZ by immunofluorescence staining. MGC803 cells were cultured in normal medium before fixation. The intracellular localization of DUB1 (green) and TAZ (red) is shown. Nuclei (blue) were stained with 4’,6-diamidino-2-phenylindole (DAPI). **B:** DUB1 and TAZ were mainly localized in the nucleus in MGC803 cells. A subcellular protein fractionation kit (Thermo Scientific, 78,840) was used for nuclear/cytoplasmic fractionation. Tubulin and histone-3 were used as the cytoplasmic and nuclear controls, respectively. **C:** The Co-IP assay revealed an association between endogenous DUB1 and TAZ in MGC803 cells. MGC803 cells were harvested with RIPA lysis buffer. Co-IP was performed using an antibody as indicated. **D-E:** DUB1 domain structure and domain deletion mutants used in the study (full length, USP domain, central domain and CTD). Full-length TAZ and TAZ deletion mutants used in the study (full length, ΔTA domain, ΔWW domain, ΔTBD). **F:** DUB1 was found to interact with TAZ through its USP domain. HEK293 cells were transfected with 2 µg of Myc-TAZ together with full-length or mutant Flag-DUB1 (full length, USP domain, central domain and CTD). After 24 h, the cells were harvested with NP-40 lysis buffer. Co-IP was performed using an anti-Myc antibody. The possible DUB1 interacting domains were detected with an anti-Flag antibody. **G:** TAZ was found to interact with DUB1 through its WW domain. HEK293 cells were transfected with 2 µg of Flag-DUB1 together with full-length or mutant Myc-TAZ (full length, ΔTA domain, ΔWW domain, ΔTBD). After 24 h, the cells were harvested with NP-40 lysis buffer. Co-IP was performed using an anti-Flag antibody. The possible DUB1 interacting domains were detected with an anti-Flag antibody. **H:** In the presence of the proteasome inhibitor MG132, the effect of DUB1 on TAZ did not result in a further increase in the TAZ protein level. MGC803 cells were transfected with 50 nM siDUB1 or siControl. After 24 h, the cells were treated with 10 mM MG132/vehicle for 6 h. Cell lysates were prepared for western blot analysis. The results are representative of three independent experiments. **I:** In the presence of the proteasome inhibitor MG132, the stabilizing effect of DUB1 on TAZ did not result in a further increase in the TAZ protein level. HEK293 cells were transfected with 0.5 µg of the Flag or Flag-DUB1 plasmid. After 24 h, the cells were treated with 10 mM MG132/vehicle for 6 h. Cell lysates were prepared for western blot analysis. The results are representative of three independent experiments. **J:** DUB1 overexpression increased the TAZ half-life in HEK293 cells. HEK293 cells were transfected with 0.5 µg of the Flag-tag or Flag-DUB1 plasmid. After 24 h, the cells were treated with 100 mM cycloheximide/vehicle for the indicated times. Cell lysates were prepared for western blot analysis. The results are representative of three independent experiments. The relative density of the TAZ protein band was measured by ImageJ software. **K:** DUB1 depletion decreased the TAZ half-life in MGC803 cells. MGC803 cells were transfected with 50 nM siControl or siDUB1. After 24 h, the cells were treated with 100 mM cycloheximide/vehicle for the indicated times. Cell lysates were prepared for western blot analysis. The results are representative of three independent experiments. The relative density of the TAZ protein band was measured by ImageJ software

We further investigated the localization of DUB1 and TAZ in gastric cancer cells. Immunostaining showed that both DUB1 and TAZ were mainly localized in the nucleus (Fig. 5A). This finding was further confirmed by a nuclear/cytoplasmic fractionation assay in multiple gastric cancer cells (Fig. 5B and Supplementary Fig. 3F-3G). Endogenous immunoprecipitation showed that DUB1 interacted with TAZ in MGC803 cells (Fig. 5C). However, we failed to detect the interaction between YAP and DUB1 (Supplementary Fig. 2C). The DUB1 protein is composed of the USP domain (deubiquitinase domain), central domain and CTD (C-terminal domain), while the TAZ protein is composed of the TB domain (TEAD binding domain), WW domain and TA domain (transcriptional activation domain) (Fig. 5D-5E). We generated the corresponding deletion constructs and further investigated the associated domain by an IP assay. The data showed that the WW domain was required for the interaction of TAZ with DUB1, while the CTD domain of DUB1 was responsible for its interaction with TAZ (Fig. 5F-5G). This conclusion was further proved via pull-down assay with purified TAZ protein and DUB1 truncated forms (Supplementary Fig. 4A). We further examined DUB1 depletion in MGC803 cells, which showed that DUB1 silencing could decrease TAZ protein level, but not mRNA level, which effect could also been found in HEK293 cells (Supplementary Fig. 3A-3D). Since TAZ is also subject to LATS phosphorylation regulation, we depleted LATS1 in MGC803 cells, which showed that DUB1 could affect TAZ protein level regardless of LATS1 existence (Supplementary Fig. 3I-3 J). DUB1 depletion decreased the TAZ protein level in MGC803 cells, and this effect was rescued by MG132 treatment (Fig. 5H). Consistent with this finding, transfection of the DUB1 overexpression plasmid into HEK293 cells showed that DUB1 increased the TAZ protein level, and this effect was minimized in the presence of the proteasome inhibitor MG132 (Fig. 5I). These data indicate that DUB1 can modulate the TAZ protein level through the proteasomal degradation system. We further tested protein stability in HEK293 cells and found that DUB1 overexpression enhanced TAZ stability (Fig. 5J). This finding was further confirmed in MGC803 cells via endogenous TAZ depletion (Fig. 5K). However, we failed to find the effect of DUB1 on YAP protein stability in both MGC803 and HEK293 cells (Supplementary Fig. 2D-2E).

### DUB1 stabilizes TAZ by inhibiting TAZ K48-linked polyubiquitination

**Fig. 6:**
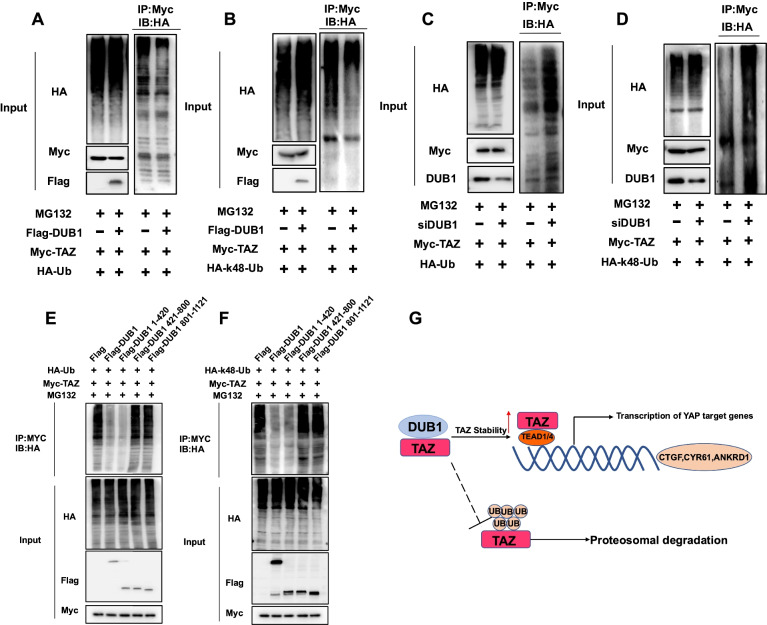
DUB1 stabilizes TAZ by inhibiting TAZ K48-linked polyubiquitination. **A:** DUB1 decreased the polyubiquitination of TAZ. HEK293 cells were transfected with 2 µg of the TAZ plasmid, 0.5 µg of the HA Ub plasmid and 0.5 µg of the Flag-tag or Flag-DUB1 plasmid. The cell extracts were subjected to immunoprecipitation with an anti-Myc antibody. Polyubiquitinated TAZ was detected via western blot analysis.**B:** DUB1 decreased K48-linked polyubiquitination of TAZ. HEK293 cells were transfected with 2 µg of the TAZ plasmid, 0.5 µg of the HA-K48-Ubi plasmid and 0.5 µg of the Flag-tag or Flag-DUB1 plasmid. The cell extracts were subjected to immunoprecipitation with an anti-Myc antibody. K48-linked polyubiquitinated TAZ was detected via western blot analysis. **C:** DUB1 depletion promoted the polyubiquitination of TAZ in MGC803 cells. MGC803 cells were transfected with 50 nM siDUB1 or siControl. After 24 h, the cells were transfected with 2 µg of the TAZ plasmid and 0.5 µg of the HA Ub plasmid. The cell extracts were subjected to immunoprecipitation with an anti-Myc antibody. Polyubiquitinated TAZ was detected via western blot analysis. **D:** DUB1 depletion promoted K48-linked polyubiquitination of TAZ in MGC803 cells. MGC803 cells were transfected with 50 nM siDUB1 or siControl. After 24 h, the cells were transfected with 2 µg of the TAZ plasmid and 0.5 µg of the HA-K48 Ubi plasmid. The cell extracts were subjected to immunoprecipitation with an anti-Myc antibody. K48-linked polyubiquitinated TAZ was detected via western blot analysis. **E:** The USP domain of DUB1 was required for its removal of polyubiquitin chains from TAZ. HEK293 cells were transfected with 2 µg of the Myc-TAZ plasmid, 0.5 µg of the HA Ub plasmid and 0.5 µg of Flag-DUB1 full-length or mutant (USP domain, central domain and CTD) plasmids. Polyubiquitinated TAZ was detected via western blot analysis. **F:** The USP domain of DUB1 was required for its removal of K48-linked polyubiquitin chains from TAZ. HEK293 cells were transfected with 2 µg of the Myc-TAZ plasmid, 0.5 µg of the HA K48-Ubi plasmid and 0.5 µg of the Flag-DUB1 full-length or mutant (USP domain, central domain and CTD) plasmids. K48-linked polyubiquitinated TAZ was detected via western blot analysis. **G:** The hypothetical model of the mechanism by which DUB1 regulates the Hippo/TAZ axis in gastric cancer. The DUB1 protein associates with TAZ and enhances TAZ protein stability by inhibiting K48-linked polyubiquitination of TAZ, which facilitates Hippo/TAZ axis activation and gastric cancer progression

Since DUB1 is a deubiquitinase, we further investigated the role of DUB1 in TAZ polyubiquitination. Ubiquitin-based immunoprecipitation in HEK293 cells showed that DUB1 overexpression reduced total TAZ polyubiquitination (Fig. 6A). Since K48-linked ubiquitination is the most common degradation signal, we examined the effect of DUB1 on K48-linked ubiquitination of TAZ. In HEK293 cells, DUB1 overexpression inhibited K48-linked ubiquitination of TAZ (Fig. 6B). However, DUB1 could also inhibit other types of ubiquitination of TAZ, which was independent of K48 site (Supplementary Fig. 4B). In MGC803 cells, depletion of endogenous DUB1 enhanced both total and K48-linked polyubiquitination (Fig. 6C-6D), while DUB1 depletion could also promote other types of ubiquitination of TAZ, which was independent of K48 site (Supplementary Fig. 4C). The in vitro ubiquitination assay also showed that DUB1 could directly facilitate the deubiquitination of TAZ, which was induced by β-TrCP (Supplementary Fig. 3E). We further investigated the domain by which DUB1 exerts its function in TAZ ubiquitination. In the ubiquitin-based immunoprecipitation assay, the USP domain (AA 1–420) of DUB1 was necessary for its deubiquitinase activity toward the TAZ protein (Fig. 6E-6F). We further investigated the precise sites of TAZ for DUB deubiquitination, since TAZ protein contains eight possible lysine sites, including K39, K45, K46, K54, K148, K157, K234 and K392. We made several mutation variants of TAZ to carry out the ubiquitination assay. The data showed that K39 and K157 sites are the most likely sites in TAZ protein for DUB1 deubiquitination (Supplementary Fig. 3E).

## Discussion

In our study, we discovered a novel deubiquitinase family member, DUB1, through a DUB siRNA library screen. An important endogenous modulator of the Hippo/TAZ axis in gastric cancer progression, DUB1 was elevated in human gastric cancer and related to poor survival in gastric cancer patients. IHC analysis showed that DUB1 expression correlated with metastasis and TAZ expression, while DUB1 depletion significantly inhibited Hippo signature gene expression on a genome-wide scale. DUB1 inhibition caused cell growth inhibition, inhibited cell cycle progression and decreased the cell invasive capacity. The molecular assays showed that DUB1 can associate with and stabilize the TAZ protein by inhibiting its K48-linked polyubiquitination in gastric cancer cells (Fig. 6G). Based on these findings, we propose that inhibition of DUB1 expression or pharmaceutical targeting of DUB1 function could be a promising strategy to suppress Hippo/TAZ-driven gastric cancers.

Conserved Hippo signaling maintains tissue hemostasis and organ size control [26]. Several studies have shown that the Hippo pathway is dysregulated in gastric cancer [27–30]. For example, the expression of the Hippo pathway effectors YAP/TAZ was found to be significantly elevated in human gastric cancer and related to poor overall survival, while depletion of YAP/TAZ or pharmaceutical targeting of the YAP/TAZ-TEAD interaction inhibited gastric cancer growth in vitro and in vivo [31]. Based on these observations, we propose that dysfunction of Hippo signaling could be one of the driver events of gastric cancer. As the Hippo pathway is an “oncogenic addiction” pathway, targeting the Hippo signaling effectors YAP/TAZ could be a plausible therapeutic strategy for gastric cancer. On the other hand, as the Hippo pathway is also an autoinhibitory pathway, the control of Hippo pathway activity depends on several internal inhibitors, such as FATs and AJUBA, and the functional MST-LATS-YAP/TAZ cascade, while abnormalities in Hippo signaling can be linked to several human cancers [32]. Several genetic studies in different malignancies have reported that genetic mutations in Hippo inhibitors, such as FATs and AJUBA, or YAP/TAZ amplification, were commonly observed [32]. However, such genetic events are rarely found in gastric cancer, possibly indicating the activation of the Hippo effectors YAP/TAZ via unknown mechanisms.

The Hippo signaling effector TAZ, which contains three domains, plays important roles in controlling Hippo signaling activity [33]. The TEAD interaction domain is responsible for its association with several transcription factors, while the WW domain mainly interacts with several TAZ modulators [34]. Due to the extensive interactions between TAZ and several transcription factors, directly targeting the TEAD-TAZ interaction becomes technically challenging. Based on the observations indicating that the TAZ protein is highly active but the inhibitory factors remain functional in gastric cancer, our research team began to target Hippo effectors by modulating their protein stability. Through screening of a DUB siRNA library, which contained more than 100 deubiquitinases, we identified DUB1 as a novel effector for TAZ stability and gastric cancer progression. This finding identified not only novel endogenous modulators of Hippo signaling but also promising targets in the Hippo/TAZ pathway in gastric cancer treatment.

In previous studies, we characterized several E3 ubiquitin ligases involved in modulating Hippo signaling effectors and cancer progression, such as RNF187 and ZNF213 [20, 24]. However, protein stability is controlled by the balance between ubiquitination and deubiquitination. Deubiquitinases, which remove ubiquitin chains from target proteins, mainly function to stabilize the target proteins [35]. Currently, more than 100 DUBs have been identified in the human genome, among which ubiquitin-specific peptidases are the largest group [36]. The DUB1 (also called USP36) gene was first discovered in HeLa cells in 2004, and the encoded protein was subsequently reported to function as a deubiquitinase through the USP domain [37]. DUB1 was found to modulate RNA helicase stability for RNA transcription in cancer cells [38]. In addition, DUB1 was reported to stabilize Myc and facilitate myc-driven cancer progression, while depletion of DUB1 could cause growth inhibition and autophagy through the P62/SQSTM1 pathway [39]. Considering these results collectively with our conclusion that DUB1 stabilizes TAZ in cancer progression, we propose that targeting DUB1 could block multiple oncogenic pathways and exert synergistic effects in cancer therapy.

## Conclusions

We identified DUB1 as an oncogene in gastric cancer both in clinical sample analysis and in experimental studies. We demonstrated that DUB1 expression was elevated in gastric carcinoma and related to poor survival. DUB1 associated with the TAZ protein and inhibited its polyubiquitination and proteasome-dependent degradation in gastric cancer cells. Our studies revealed a novel function of DUB1 in Hippo signaling at multiple levels. As DUB1 is a novel modulator of Hippo signaling, modulation of its activity or gene expression level could be an appealing strategy to treat gastric cancer.

## Supplementary Information

**Additional file 1.** 

**Additional file 2.** 

**Additional file 3.** 

**Additional file 4.**

**Additional file 5.** 

**Additional file 6.** 

**Additional file 7.** 

## Data Availability

The publicly available data are provided in GEO database. The original siRNA screening data are provided in supplementary materials. The original data for WB and QPCR are provided in supplementary materials. The cell line authentications are shown in supplementary materials. The approved document of human sample ethnics is shown in the supplementary materials.

## Abbreviations

DUB: Deubiqutinase
USP: Ubiquitin specific peptidese
TAZ: Transcriptional Co-Activator With PDZ-Binding Motif
PFS: Progression-free survival
EdU: 5-Ethynyl-2’-deoxyuridine
GSEA: Gene set enrichment analysis
IHC: Immunohistochemistry
