# Supplementary figures and images for: DUB1 suppresses Hippo signaling by modulating TAZ protein expression in gastric cancer
Source: J Exp Clin Cancer Res. 2022 Jul 12;41:219. doi: 10.1186/s13046-022-02410-5 (PMC9275142; doi:10.1186/s13046-022-02410-5)

Supplementary Figure1

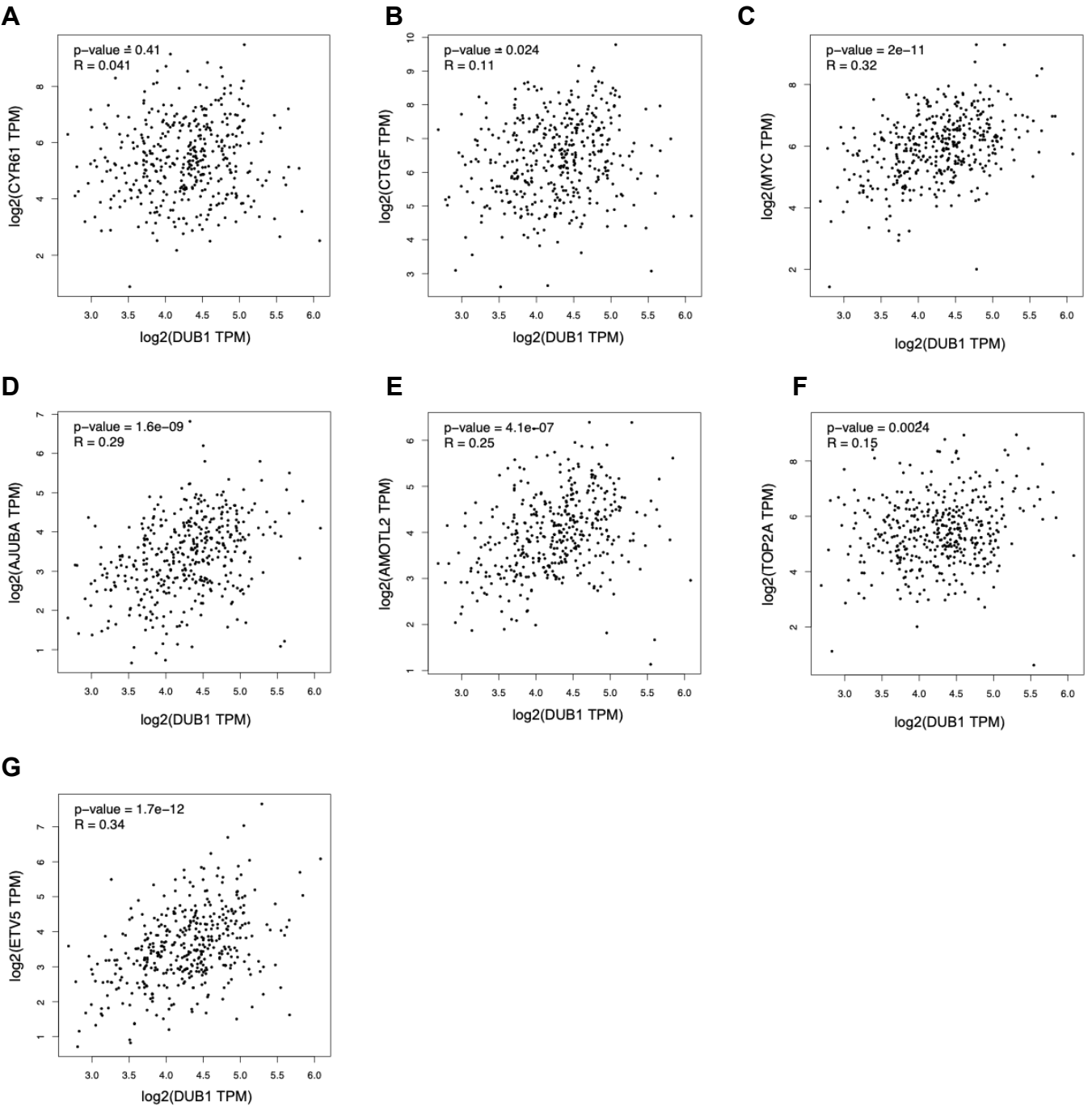

Supplement: Supplementary file 1 — Additional file 1. [file 13046_2022_2410_MOESM1_ESM.pdf]

Supplementary Figure2

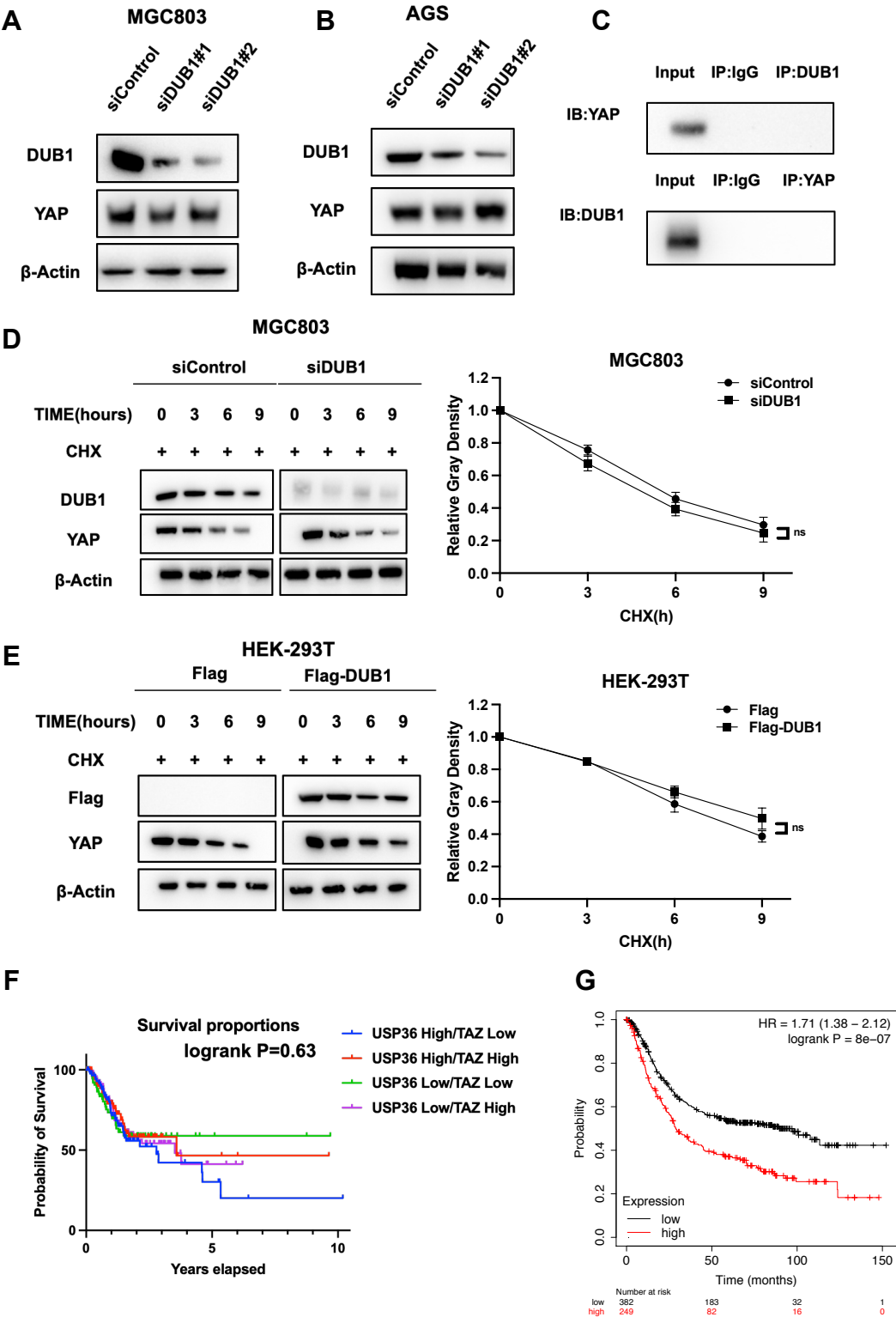

Supplement: Supplementary file 2 — Additional file 2. [file 13046_2022_2410_MOESM2_ESM.pdf]

Supplementary Figure3

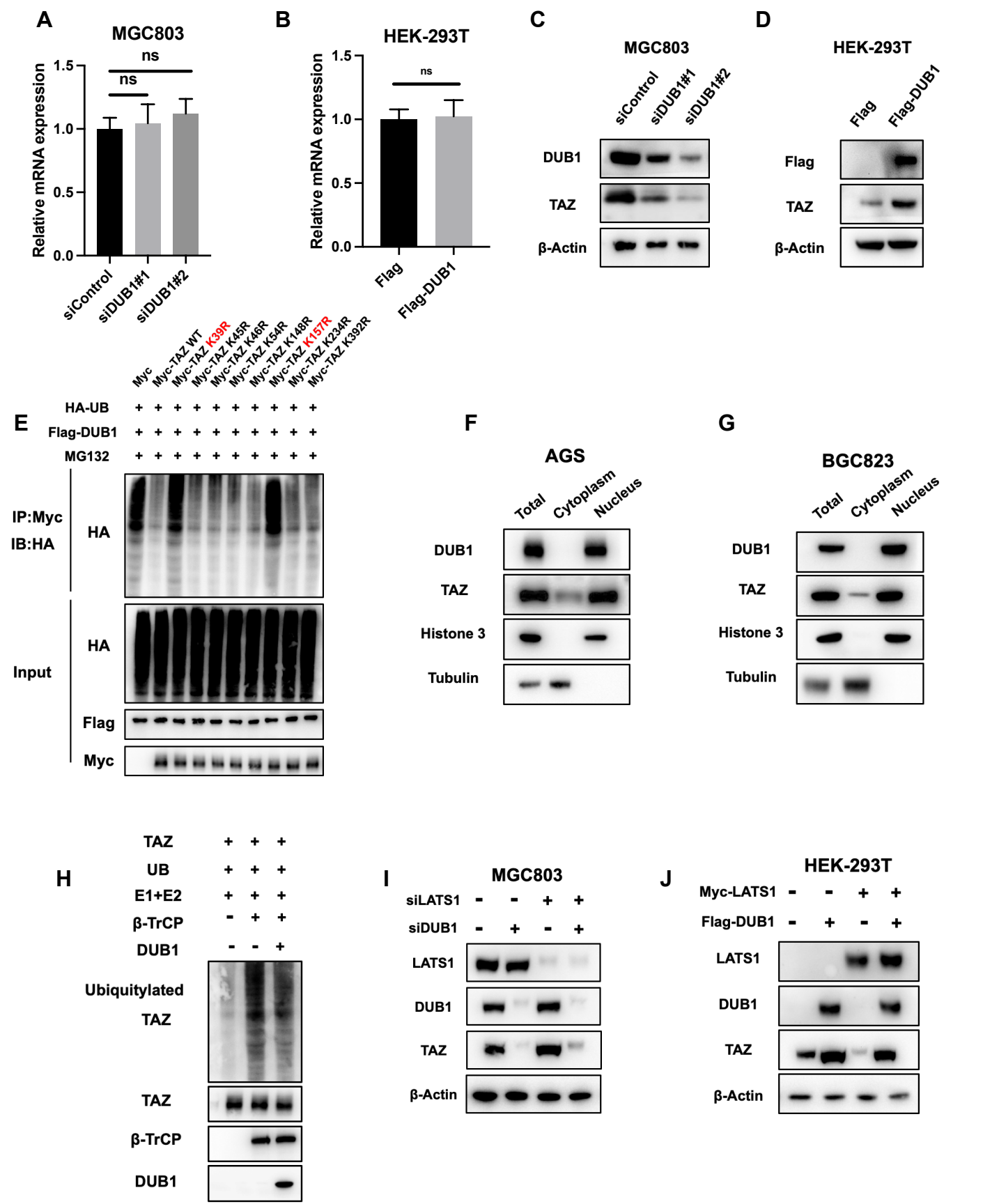

Supplement: Supplementary file 3 — Additional file 3. [file 13046_2022_2410_MOESM3_ESM.pdf]

Supplementary Figure 4

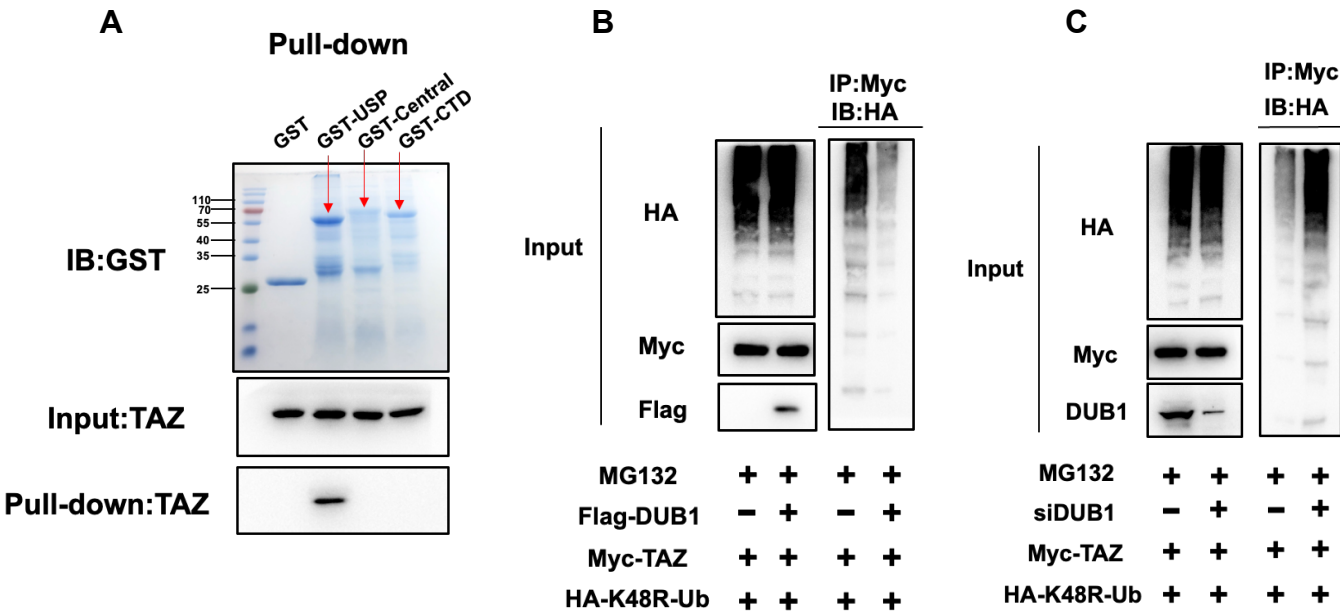

Supplement: Supplementary file 4 — Additional file 4. [file 13046_2022_2410_MOESM4_ESM.pdf]
