# Supplementary material for: DUB1 suppresses Hippo signaling by modulating TAZ protein expression in gastric cancer
Source: J Exp Clin Cancer Res. 2022 Jul 12;41:219. doi: 10.1186/s13046-022-02410-5 (PMC9275142; doi:10.1186/s13046-022-02410-5)
Supplement: Supplementary file 7 — Additional file 7. [file 13046_2022_2410_MOESM7_ESM.pdf]

CTGF		CTGF
control	0.9266595	control
control	1.120494	control
USP1	0.3569116	USP36
FBXO1	0.4455766	USP37
USP16	0.4471422	UCHL3
USP30	0.4483284	ATXN3
OTUD5	0.4501485	USP17L2
USP9X	0.4509854	USP20
PAN2	0.4804577	UCHL5
USP53	0.4818221	USP38
TNFAIP	0.4886988	USP40
OTUD7A	0.4888125	OTUD4
FBXO8	0.5048133	CYL0
USP29	0.5183401	USP24
USP12	0.5425176	USP11
USP50	0.5549464	USP39
USP7	0.5688184	USP8
USP41	0.5706465	UBTD1
OTUB1	0.5766439	USPL1
UCHL1	0.5802543	STAMBP
USP34	0.5924227	USP14
SENP2	0.6124843	USP3
OTUB2	0.6206439	USP42
USP9Y	0.6243423	UBTD2
PSMD14	0.6365459	UFD1L
COPS5	0.6682351	JOSP2
VCPIP1	0.715036	USP35
USP51	0.7177723	OTUD1
USP21	0.7342152	USP27X
USP18	0.7349314	USP4
MPND	0.7354694	USP25
USP2	0.7589731	UBL3
USP13	0.8023474	USP43
UBR1	0.8035231	USP47
USP46	0.8084161	PRPF8
YOD1	0.873441	USP54
BAP1	1.072662	UBL5
USP19	1.145582	BRCL3
USP28	1.169398	USP33

USP49	1.188868	ZRANB1
UEVLD	1.643048	USP5
USP45	2.244599	USP44
USP48	2.263998	STAMBPL1
OTUD7B	2.317117	USP22
OTUD6B	3.378936	UCK2
JOSD1	6.336721	USP6
USP10	7.003222	USP31
UBL4A	9.815673	USP15
